# Role of fenofibrate in multiple sclerosis

**DOI:** 10.1186/s40001-024-01700-2

**Published:** 2024-02-09

**Authors:** Ahmad A. Abulaban, Hayder M. Al-kuraishy, Ali I. Al-Gareeb, Engy Elekhnawy, Asma Alanazi, Athanasios Alexiou, Marios Papadakis, Gaber El-Saber Batiha

**Affiliations:** 1 College of Medicine, King Saud Bin Abdulaziz University for Health Sciences, Riyadh, Saudi Arabia; 2 Division of Neurology, King Abdulaziz Medical City, Ministry of the National Guard Health Affairs, Riyadh, Saudi Arabia; 3 King Abdullah International Medical Research Center, Riyadh, Saudi Arabia; 4 Department of Pharmacology, Toxicology and Medicine, College of Medicine, Mustansiriyah University, Baghdad, 14132 Iraq; 5 Pharmaceutical Microbiology Departments, Faculty of Pharmacy, Tanta University, Tanta, 31527 Egypt; 6 College of Medicine, King Saud Bin Abdulaziz University for Health Sciences (KSAU-HS), Riyadh, Saudi Arabia; 7 King Abdullah International Medical Research Center (KAIMRC), Riyadh, Kingdom of Saudi Arabia; 8 University Centre for Research & Development, Chandigarh University, Chandigarh-Ludhiana Highway, Mohali, Punjab India; 9 Department of Research & Development, Funogen, Athens Greece; 10 Department of Research & Development, AFNP Med, 1030 Vienna, Austria; 11 Department of Science and Engineering, Novel Global Community Educational Foundation, Hebersham, NSW 2770 Australia; 12 Department of Surgery II, University Hospital Witten-Herdecke, Heusnerstrasse 40, University of Witten-Herdecke, 42283 Wuppertal, Germany; 13 Department of Pharmacology and Therapeutics, Faculty of Veterinary Medicine, Damanhour University, Damanhour, 22511 AlBeheira Egypt

**Keywords:** Multiple sclerosis, Demyelination, Oxidative stress, Fenofibrate

## Abstract

Multiple sclerosis (MS) is the most frequent inflammatory and demyelinating disease of the central nervous system (CNS). The underlying pathophysiology of MS is the destruction of myelin sheath by immune cells. The formation of myelin plaques, inflammation, and injury of neuronal myelin sheath characterizes its neuropathology. MS plaques are multiple focal regions of demyelination disseminated in the brain's white matter, spinal cords, deep grey matter, and cerebral cortex. Fenofibrate is a peroxisome proliferative activated receptor alpha (PPAR-α) that attenuates the inflammatory reactions in MS. Fenofibrate inhibits differentiation of Th17 by inhibiting the expression of pro-inflammatory signaling. According to these findings, this review intended to illuminate the mechanistic immunoinflammatory role of fenofibrate in mitigating MS neuropathology. In conclusion, fenofibrate can attenuate MS neuropathology by modulating different pathways, including oxidative stress, autophagy, mitochondrial dysfunction, inflammatory-signaling pathways, and neuroinflammation.

## Introduction

Multiple sclerosis (MS) is the most frequent inflammatory and demyelinating disease of the central nervous system (CNS) [1]. MS disrupts motor and sensory neuronal signal transmission, leading to motor and sensory deficits. It is characterized by symptoms, including vision loss in one eye, double vision, muscle weakness, and motor-sensory incoordination [2]. MS patients may have a prodromal phase characterized by cognitive impairments, and neuropsychiatric symptoms continue for years before the manifestation of MS symptoms. Clinical presentations of MS are motor, sensory, and autonomic dysfunctions. A specific feature of MS depends on the site of lesions in the CNS, including visual loss due to optic neuritis, muscle spasm, hyperreflexia due to spinal cord injury, ataxia due to cerebellar involvement and motor-sensory incoordination [3, 4]. Notably, 85% of MS patients presented with acute exacerbations, and 15% of MS patients presented with gradual motor-sensory dysfunction without a period of recovery [5–7]. The clinical spectrum of MS includes CIS, RRMS, and SP/PPMS. According to Lublin, there are two main MS phenotypes: relapsing and progressive, additionally modified by the presence or absence of activity-relapses and new changes in MRI (i.e., RRMS active or inactive/stable, SP/PPMS active or inactive) [8–10].

MS may be progressive over time or relapsing forms in which the symptoms disappear and return. It has been reported that about one million people in USA will be affected by MS in 2022 [5]. MS affects about 2.8 million people worldwide [6]. MS is more common in women at 20–50 years [7]. Of note, MS was initially identified by Jean-Martin Charcot, a French neurologist, in 1868, who described multiple scars in the brain and spinal cord [8]. To better understand the MS pathophysiology, we focused on the lipids that play an influential role in the disease background. Lipids are not only considerably involved in the formation of myelin sheath but are also involved in cell signaling, communication, and in transport in the CNS. Thus, lipids seem probable candidates for processes underlying the active and progressive phase of MS and potential targets for new, effective, and stage-specific therapeutic interventions [9–12]. Previous studies illustrated that peroxisome proliferative activated receptor alpha (PPAR-α) agonists such as fenofibrate, gemfibrozil and ciprofibrate could attenuate the inflammatory reactions in MS. It has been reported that gemfibrozil attenuates experimental autoimmune encephalomyelitis (EAE), an animal model of relapsing–remitting multiple sclerosis (RMS) in mice by inhibiting encephalitogenic of myelin basic protein (MBP)-primed T cells and switched the immune response from a Th1 to a Th2 profile independent of PPAR-α [13–16]. Likewise, ciprofibrate was proposed to be effective in different autoimmune diseases, including MS, by increasing the production of anti-inflammatory cytokines, inhibiting T cells specific for MBP and reducing microglial activity [16].

Oral administration of gemfibrozil, ciprofibrate, and fenofibrate repressed clinical signs of EAE by shifting the cytokine secretion of human T-cell lines by inhibiting interferon-gamma (IFN-γ) and promoting IL-4 secretion [17]. Similarly, fenofibrate inhibits differentiation of Th17 by inhibiting the expression of pro-inflammatory signaling [18–20]. These outcomes propose that PPARα agonists may be attractive nominees for use in human inflammatory conditions, such as MS. Compared with gemfibrozil, fenofibrate produced significantly greater reductions in total cholesterol, triglycerides and significantly more significant increases in high-density lipoprotein (HDL). However, fenofibrate is less effective compared to new-generation pemafibrate in amelioration of lipid profile [18]. Moreover, fenofibrate has pleiotropic anti-inflammatory and antioxidant effects that may reduce inflammatory and oxidative stress disorders in different autoimmune disorders, as in MS [17]. According to these findings, this review aimed to clarify the mechanistic immunoinflammatory role of fenofibrate in mitigating MS neuropathology.

## Pathophysiology of MS

The underlying pathophysiology of MS is the destruction of myelin sheath by immune cells or failure in the production of myelin [4]. The characteristic feature of MS neuropathology is the formation of myelin plaques, inflammation, and injury of neuronal myelin sheath [9]. Myelin plaque represents a clustering of inflammation, myelin breakdown, astrogliosis, oligodendrocyte injury, neurodegeneration, axonal loss, and remyelination [9]. Breakdown of immune response and regulation due to environmental factors and genetic predisposition induce MS neuropathology. Abnormal immune response in genetically susceptible subjects to some environmental factors triggers cell-mediated immunity with the development of demyelination [9].

Of interest, MS is regarded as a hereditary disease, though different genetic variations may increase MS risk [10–12]. Different environmental factors trigger the development of MS, including early exposure to infectious agents, which attenuate MS risk. Epstein–Barr virus (EBV), which causes infectious mononucleosis, is implicated in the pathogenesis of MS by 32 folds [14]. Besides, smoking, organic solvents, obesity and certain diets may increase MS risk [15, 18]. However, gout and hyperuricemia are protective against the development of MS [17].

CNS plaques in MS mainly affect the brain stem, basal ganglia, optic nerve, and spinal cord, though peripheral neurons are rarely affected [18, 19]. In the MS plaques, the inflammatory profile is characterized by infiltration of immune cells, including T lymphocytes, monocytes, B and plasma cells [20]. Three different types of MS plaques have been revealed, including type I (macrophages and T lymphocytes dominate lesions), type II (have additional accumulation of activated complement and immunoglobulins) and type III (in which there is additional apoptosis of oligodendrocytes and glial cells) [21, 22]. MS lesions seem identical in the affected patient but vary among patients, reflecting different stages of MS progression rather than different disease subtypes [23]. In MS, oligodendrocytes involved in the myelin sheath synthesis are mainly affected [24]. Myelin sheath is involved in generating action potential and transmitting electrical signals. Progressive loss of myelin sheath with axonal injury leads to neuronal dysfunction [25]. Partial remyelination may occur during the remission state, and demyelination is returned during the relapse state. These changes promote plaque formation in the multiple sites in the CNS. In addition, reactive astrocytosis in response to neuronal injury promote plaque formation [26].

**Fig. 1:**
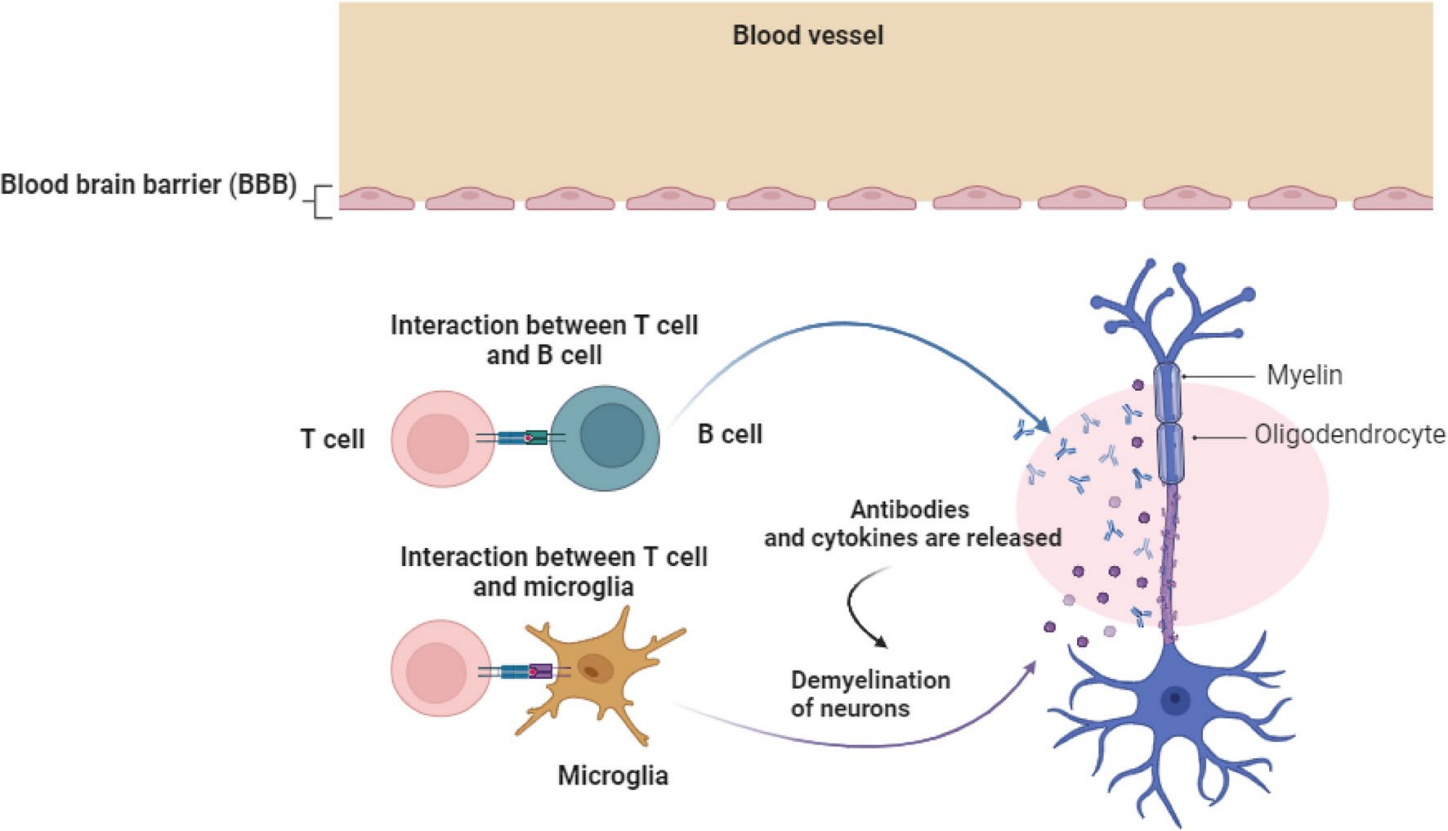
Pathophysiology of MS

On the other hand, inflammation plays an integral role in the pathogenesis of MS due to the uncontrolled activation of T lymphocytes [27]. Peripheral auto-reactive T lymphocytes trigger inflammatory changes in the MS [28]. However, the underlying mechanism for activating peripheral auto-reactive T lymphocytes is poorly identified. It has been shown that polyclonal activation of peripheral auto-reactive T lymphocytes by viral and bacterial antigens or molecular mimicry could be the possible mechanism [29]. Peripheral auto-reactive T lymphocytes can cross blood–brain barrier (BBB) through binding integrins on the immune cells and VCAM-1 on the endothelial cells [30–33]. Following entry of peripheral auto-reactive T lymphocytes, these cells bind MHCII expressed by dendritic and antigen-presenting cells, leading to disruption of myelin components and release of other CNS antigens with subsequent recruitment of other immune cells and production of specific myelin autoantibodies, which promote further injury and loss of myelin sheath [34–36]. The interaction between auto-reactive T lymphocytes and myelin antigens triggers the release of pro-inflammatory and inflammatory cytokines with the production of antibodies [36]. These immune-inflammatory reactions cause further injury of BBB that promotes entry of auto-reactive T lymphocytes and generation of soluble factors which attack synaptic regions, causing neuronal dysfunction [35, 36]. These neuropathological changes lead to progressive loss of myelin sheath and axonal damage (Fig. 1).

Moreover, neurodegeneration is also intricate in MS neuropathology. Mounting evidence highlighted that neurodegeneration occurs early in both gray and white matter of MS patients [37–39]. In addition, axonal degeneration analysis in chronic inactive lesions of secondary progressive MS patient spinal cords indicated a 61% reduction in axonal density [40]. Interestingly, gray matter atrophy rates have been correlated with worsening disability in MS. One of the mechanisms hypothesized to explain the diffuse neurodegeneration found in MS patients involves mitochondrial dysfunction [40–43]. Diffuse mitochondrial dysfunction secondary to MS induces inadequate energy production and intracellular dysregulation. This dysfunction impairs anterograde and retrograde transportation along axons.

Furthermore, progressive inflammation and autoimmunity trigger neurodegeneration in MS. Taken together, autoimmunity, demyelination, inflammation, and neurodegeneration are involved in the pathogenesis of MS. Different studies revealed that fenofibrate is effective in reducing neurodegeneration in patients with diabetic retinopathy [5, 43–45] and experimental Parkinson disease (PD) model by inhibiting neuroinflammation and oxidative stress. In addition, fenofibrate has potent anti-inflammatory effects through direct inhibition of pro-inflammatory cytokines or indirectly by reducing dyslipidemia-induced inflammation. Furthermore, fenofibrate can reduce autoimmunity and demyelination in MS through modulation of Th1/Th2 immune response. Thus, fenofibrate can modulate the components of MS neuropathology.

Acute attack of MS is treated by corticosteroids [43], and plasmapheresis is indicated when treatment with corticosteroid is ineffective. Chronic MS is managed by disease-modifying treatments, such as interferons, glatiramer, and mitoxantrone [45, 46]. However, lipid-based therapeutics linked with possible future-based therapeutic interventions such as fenofibrate are proposed in the management of MS [54, 55].

## Lipid dysregulation in MS

Furthermore, lipid dysregulation is associated with MS neuropathology as lipid molecules play a dual role in MS, both as target molecules of myelin destruction and as mediators of inflammation [56–58]. Altered lipid metabolism with systemic inflammation may contribute to immune activation. Evidence suggests that abnormalities in the lipid-binding proteins of myelin and sphingolipid content that confers increased immunogenicity may cause the autoimmune response against the myelin sheath [59]. CNS is, after all, the second organ richer in lipid content after adipose tissue. In addition, soluble factors secreted by adipose tissue modulate inflammatory responses and contribute to metabolic dysfunction, which may be important in MS pathophysiology. Inflammatory cytokines participate in proatherogenic changes in lipid metabolism by reducing HDL levels and impairing anti-inflammatory and antioxidant functions.

Consequently, the protective actions of HDL can be limited in chronic inflammatory diseases, such as MS [60–63]. A case–control study illustrated that dysfunctional HDL is correlated with inflammatory mediators in MS patients. Fenofibrate improves circulating HDL and reduces inflammatory disorders. Dyslipidemia with low HDL and high triglyceride (TG) correlated disease activity and disability in MS patients. It remains to be elucidated whether altered lipid metabolism contributes to harmful immune response, possibly through inflammation, or is secondary to neurological disability in MS. A cohort study involving 492 MS patients revealed that serum lipid profile has modest effects on disease progression in MS. Worsening disability is associated with higher levels of LDL, total cholesterol and triglycerides. Higher HDL is associated with lower levels of acute inflammatory activity [63–66]. Fenofibrate has a potent effect in regulating lipid profiles mainly TG, HDL and LDL. These observations indicated that dysregulation of lipid profile triggers abnormal immune response and increases the risk of autoimmunity as in MS. Fenofibrate through modulation of blood lipid can mitigate the detrimental effects of TG and LDL on MS neuropathology.

## Potential beneficial effects of fenofibrate in MS

### Pharmacology of fenofibrate

**Fig. 2:**
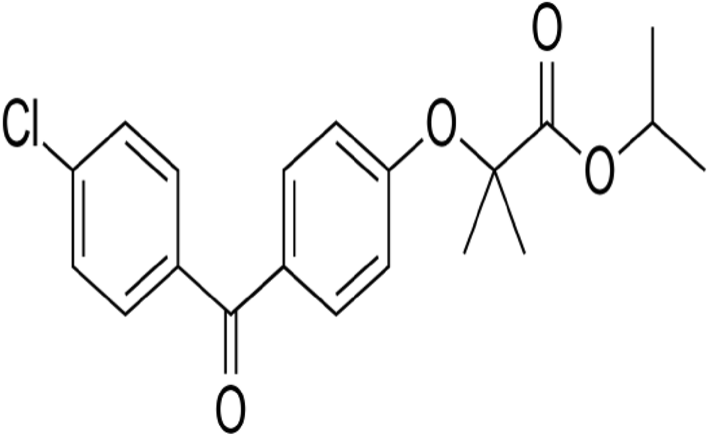
Chemical structure of fenofibrate

Fenofibrate is a chlorobenzophenone derivative drug (Fig. 2) used to manage hypertriglyceridemia and mixed hyperlipidemia [50, 51].

Fenofibrate was initially synthesized from clofibrate in France in 1974; it was known as precetofen, which was renamed fenofibrate according to the WHO no-proprietary guideline [49]. Fenofibrate acts via activation of PPAR-α, leading to activation of lipoprotein lipase and reduction of apolipoprotein CIII, resulting in lipolysis and elimination of TG from plasma. Fenofibrate increases expression of apolipoprotein AI and AII, leading to reducing levels of LDL and VLDL with increment of HDL [50, 51]. Of interest is that fenofibrate use has been shown to be effective in managing diabetic retinopathy [52]. In addition, fenfibrate use in T2DM patients reduces the risk of amputation by 37% regardless of glycemic control [53]. Fenofibrate is subjected to drug interaction with bile acid sequestrants, immunosuppressant agents, warfarin, and statins. The most common adverse effects of fenofibrate are myalgia, headache, arthralgia, and renal stone. Fenofibrate is contraindicated in patients with renal impairment, liver dysfunction, gallbladder diseases, hypothyroidism, and hypersensitivity [50, 51]. Due to its anti-inflammatory and antioxidant effects, fenofibrate was suggested for treating different neurodegenerative disorders. PPAR-α is expressed in the brain and other organs and plays a significant role in oxidative stress, energy homeostasis, mitochondrial fatty acids metabolism and inflammation. PPAR-α contributes to the regulation of genes coding proteins that are intricate in glutamate homeostasis and cholinergic/dopaminergic signaling in the brain.

Furthermore, PPAR-α regulates the expression of genes coding enzymes engaged in amyloid precursor protein (APP) metabolism. It activates gene coding of α secretase, which is responsible for the non-amyloidogenic pathway of APP degradation [67–69]. It also down-regulates β secretase (BACE-1), the main enzyme responsible for amyloid beta (Aβ) peptide release in AD. In AD the expression of genes of PPAR-α and PPAR-γ coactivator-1 alpha (PGC-1α) is significantly decreased. PPARs are altered not only in AD but in other neurodegenerative/neurodevelopmental and psychiatric disorders [69–71]. PPAR-α downregulation may decrease anti-oxidative and anti-inflammatory processes. It could be responsible for the alteration of fatty acid transport, lipid metabolism and disturbances of mitochondria function in the brain of AD patients. Specific activators of PPAR-α may be important for the improvement of brain cell metabolism and cognitive function in neurodegenerative and neurodevelopmental disorders [71]. However, the potential mechanism of fenofibrate in MS is not fully elucidated.

### Role of fenofibrate in MS

PPAR-α agonists have been used over decades to treat human metabolic disorders with little toxicity, making them an attractive candidate for use in the management of MS. PPAR-α agonists, such as fenofibrate, can alter the phenotype of myelin autoreactive T cells and their encephalitogenicity [62, 72].

It has been shown that PPAR-α is expressed in T cells, and its ligands inhibit T-cell proliferation, release of IL-2 and regulation of inflammatory response [63]. Fenofibrate can attenuate autoimmune response in mice with experimental Sjogren syndrome through modulation of T-cell's immune response. PPAR-α-deficient mice had abnormal immune responses to inflammatory mediators, such as prostaglandins and leukotrienes. Expression of adhesion molecules, cyclooxygenase-2 (COX-2) and IL-6 are inhibited by PPAR-α ligands [65]. PPAR-α ligands inhibit expression of NF-κB through increasing expression of NF-κB inhibitor (IκBα) [66]. PPAR-α ligand WY14643 blocks IgG interaction with myelin oligodendrocyte in mice [66]. PPAR-α ligands such as fenofibrate have anti-inflammatory effects by increasing the release of anti-inflammatory cytokines [67]. Interestingly, PPAR-α ligands promote Th2 cytokine production [68].

It has been revealed that fenofibrate can reduce the inflammatory reactions in MS through inhibition expression of IL-2 in lipopolysaccharide (LPS)-induced microglia activation [47]. Moreover, fenofibrate inhibits microglial expression of CD14, which plays a critical role in TLR signaling, signifying a mechanism by which fenofibrate suppresses the release of pro-inflammatory molecules [47]. Prominently, fenofibrate suppression of EAE was associated with decreased expression of IL-12 family cytokine mRNAs and mRNAs encoding TLR4, CD14, and MyD88 known to play critical roles in MyD88-dependent TLR signaling [47]. These findings propose that fenofibrate may modulate the development of EAE by inhibiting the production of IL-12 and MyD88-dependent-signaling pathway [47].

Fenofibrate has the ability to inhibit differentiation of Th17 significantly compared to other PPAR-α agonists, such as bezafibrate. Fenofibrate blocks IL-21 and STAT3 expression is required for Th17 differentiation [48]. It has illustrated that fenofibrate mitigates non-lipid-associated autoimmune diseases, such as autoimmune colitis and MS [48]. It has been hypothesized that fenofibrate reduces the differentiation of Th17 cells and inhibits transforming growth factor-*β* (TGF-*β*) and IL-6-induced differentiation of Th17 cells*.* However, other PPAR*α* agonist's bezafibrate did not affect Th17 differentiation, indicating that this effect of fenofibrate might be PPAR*α* independent [48]. A case–control study revealed that prolonged use of fenofibrate reduces inflammatory biomarkers, including IL-6 and CRP, in patients with dyslipidemia [54], signifying possible fenofibrate effects on systemic inflammation. Janssen et al. [55] observed that IL-6 is involved in the pathogenesis of MS by activating T cells [55]. It has been observed that fenofibrate and other PPAR-α agonists activate the neurons' myelination by increasing expression of sterol regulatory element binding factors (SREBF), which increase sterol biosynthesis [56]. PPARα can stimulate SREBF signaling via multiple mechanisms, including increasing SREBF expression, enhancing SREBF proteolytic cleavage, and increasing SREBF activity through the recruitment of transcriptional co-activators [56]. Gemfibrozil activates PPARα and increases the expression of myelin in human oligodendrocytes. PPARα activation can also stimulate SREBF signaling. Fibrates have been considered as potential therapeutics for diseases associated with impaired oligodendrocytes, such as MS, adrenoleukodystrophy, and traumatic brain injury [56]. Therefore, fenofibrate and gemfibrozil promote myelination by activating SREBFs in MS. Of note, SREBFs are reduced in MS [57]. Fenofibrate and other PPAR-α agonists have been shown to inhibit microglia are involved in MS neuropathology [58].

Collectively, fenofibrate could be effective in managing MS by its anti-inflammatory effect and modulation of SREBFs. Accordingly, fenofibrate regulates two important aspects, including neuroinflammation and neurodegeneration, which are highly intricate in MS neuropathology.

### Effect of fenofibrate on neuroinflammation in MS

Neuroinflammation is associated with the progression of different neurodegenerative disorders. T and B cells in the CNS trigger inflammatory disorders and the development of neuroinflammation. Neuroinflammation in the early stage of MS can cause synaptopathy independent of the demyelination process, and this may explain cognitive dysfunction in the early phase of MS patients [73]. In the late phase of MS, exaggeration of immune disturbance and development of neuroinflammation promote MS pathogenesis. It has been shown that cholinergic activity is reduced in MS patients, which regulates the activity and response of immune cells. Reduction of acetylcholine level in the immune cells promotes the release of pro-inflammatory cytokines with the development of neuroinflammation [74, 76]. Therefore, attenuation of neuroinflammation could be a therapeutic strategy in the mitigation of MS neuropathy.

Different preclinical studies revealed a potential role of fenofibrate against the development and progression of neuroinflammation. Fenofibrate inhibits neuroinflammation in traumatic brain injury by suppressing oxidative stress in rats. Fenofibrate has a neuroprotective effect against the development and progression of MS by inhibiting mitochondrial dysfunction, oxidative stress, and neuroinflammation that contribute mutually to neurodegeneration [77]. The underlying mechanism for fenofibrate role against the development of neuroinflammation is related to the inhibition of inflammatory-signaling pathway, antioxidant and anti-inflammatory effects. Remarkably, fenofibrate activates neuronal nicotinic cholinergic receptors with subsequent inhibition of neuroinflammation [78]. These findings indicated that fenofibrate may reduce MS pathogenesis by modulating neuroinflammation.

Toll-like receptors (TLRs) are innate immune sensors that alert the immune system to the presence of external pathogens [79, 80]. Activation of TLR triggers the release of pro-inflammatory cytokines and activation of adaptive immune response to eliminate invading pathogens. TLR can detect danger signals, which are products of inflammation and tissue injury. TLRs are highly expressed by immune cells in the CNS and are involved in the MS neuropathology [81]. Of note, TLR agonists participate in the amplification of harmful inflammatory responses. It has been established that PPAR-α agonists have reciprocal interactions with TLRs, as activation of PPAR-α inhibits expression of TLRs via several mechanisms. Fenofibrate inhibits the expression of CD14, which increases the expression of TLR and the release of pro-inflammatory cytokines. In addition, fenofibrate inhibits the release of IL-12 and the expression of the MyD88–TLR4-signaling pathway [47]. Therefore, PPAR-α agonist fenofibrate can inhibit the primary immune response in MS neuropathology by inhibiting TLRs and their effectors.

Furthermore, different inflammatory-signaling pathways, including NF-κB and nod-like receptor pyrin three receptor (NLRP3) inflammasome, are involved in the pathogenesis of MS [82–87]. NF-κB is a DNA-binding protein necessary for transcription of chemokines and pro-inflammatory cytokines. Particularly, immune deregulation encourages the commencement of NF-κB with consequential neuronal injury, neuroinflammation, and development of neurodegeneration [88–91]. NLRP3 inflammasome is involved in the activation of caspase-1 and maturation of IL-1β and IL-18 [92–94]. Diverse stimuli including NF-κB trigger NLRP3 inflammasome. NLRP3 inflammasome is intricate in the pathogenesis of neuroinflammation and development of neurodegeneration [95–100]. NF-κB is exaggerated in MS, leading to immune dysregulation and induction release of pro-inflammatory cytokines. Inhibition of the NF-κB-signaling pathway by teriflunomide, fingolimod and dimethyl fumarate may reduce MS severity [100–102]. Chen et al. [103] observed that native and memory B cells from MS patients have a higher level of phosphorylated NF-κB, which was inhibited by mycophenolate. In addition, glatiramer attenuates the activation of NF-κB by CD40, which is over-activated in MS [103]. Likewise, NLRP3 inflammasome is also exaggerated and linked with the severity of MS [104]. NLRP3 inflammasome within activated microglia promotes the expression and release of IL-1β and IL-18. Evidence from preclinical and clinical findings illustrated that aberrant activation of NLRP3 inflammasome is associated with the pathogenesis of MS [104]. Over-activation of NLRP3 inflammasome in MS is evident by increasing IL-1β CSF levels in severely affected patients [105]. Targeting NLRP3 inflammasome by specific inhibitors can reduce MS severity [105].

Fenofibrate has a potent anti-inflammatory effect against the development of pulmonary inflammation by inhibiting the expression of NF-κB and NLRP3 inflammasome [106]. Notably, fenofibrate prevents retinal injury and disruption of retinal blood barrier by inhibiting the NF-κB-signaling pathway [107]. Besides, fenofibrate can reduce diabetic retinopathy by inhibiting the expression of NLRP3 inflammasome [108–111]. Thus, fenofibrate might effectively reduce MS pathogenesis by targeting the most common inflammatory-signaling pathways, including NF-κB and NLRP3 inflammasome [112–116].

Interestingly, TLRs, the release of pro-inflammatory-signaling pathways and inflammatory mediators are involved in the development and progression of neuroinflammation in MS. Inhibition of TLRs and release of pro-inflammatory by fenofibrate result in momentous suppression of neuroinflammation in MS [117–121].

### Effect of fenofibrate on neurodegeneration in MS

It has been illustrated that mitochondrial dysfunction and deregulation of neuronal energy balance secondary to MS induce inadequate ATP production and intracellular dysregulation [122–126]. This dysfunction impairs anterograde and retrograde transportation along axons, leading to progressive neurodegeneration in MS [125–127]. Moreover, mitochondrial dysfunction contributes to the loss of neurons and axons in MS due to uncontrolled activation of microglia and associated neuronal injury [80]. Impairment of mitochondrial permeability transition pore by Ca^2+^ dyshomeostasis and ROS is the central mechanism for the development of mitochondrial dysfunction in MS [81]. Pathological opening of mitochondrial permeability transition pore in response to nitrogen species, Ca^2+^ and ROS, induces an influx of many solutes into the mitochondrial matrix, leading to matrix expansion and mitochondrial rupture with eventual cell deaths [81]. Merlini et al. [82] revealed that mitochondrial dysfunction is regarded as an essential trigger of programmed axon death in MS. Uric acid and serum lactate are considered as potential biomarkers of mitochondrial dysfunction [83]. A case–control study that included 32 MS patients, and 20 healthy controls showed that lactate serum level but not serum uric acid was increased in MS patients compared to the controls [83]. It has been proposed that mitochondrial dysfunction alters lymphocyte homeostasis, leading to a defective apoptotic process of auto-reactive T cells, allowing them to perpetuate within the CNS and continue the inflammation cycle in MS patients [84]. Activation of Th1 cells and their lymphokines, such as interferon-gamma (INF-α) and IL-2, which induce the transformation of B-lymphocytes to plasma cells produce autoantibodies against myelin antigens [84]. Therefore, mitochondrial dysfunction could be a primary cause for MS progression through alteration of lymphocyte activity, or a secondary outcome due to oxidative stress caused by MS. Thus, mitigation of mitochondrial dysfunction could be effective mechanistic way to prevent the progression of MS.

On the other side, PPAR-α agonists have an important role in the modulation of mitochondrial function in diabetic patients [85]. Of interest, fenofibrate improves insulin sensitivity by enhancing mitochondrial β-oxidation [86]. In a similar way, fenofibrate inhibits mitochondrial dysfunction in burn patients [86]. In addition, fenofibrate enhances neurogenesis via modulation of mitochondrial biogenesis in experimental ischemic reperfusion injury [87]. The protective effect of fenofibrate against the development of mitochondrial dysfunction is mediated by increasing the expression of mitochondrial uncoupling protein two, which protects mitochondria from the harmful oxidative stress by reducing the generation of ROS [88]. However, a higher concentration of fenofibrate may induce the development of mitochondrial dysfunction via inhibition of mitochondrial respiratory chain complex I [89]. Therefore, the appropriate dose of fenofibrate could be effective against MS through the modulation of mitochondrial dysfunction.

In addition, oxidative stress plays an integral role in the pathogenesis of MS via the enhancement of the demyelination process and neurodegeneration [73]. ROS promotes peripheral activation of T cells and the development of autoreactive T cells. ROS triggers microglia activation and induces neuronal apoptosis [73]. Inflammatory reactions in MS can provoke oxidative stress bursts in the activated macrophages and microglia, leading to neuronal demyelination. In turn, oxidative stress and released ROS enhance the propagation of inflammation and neurodegeneration in MS [74]. Therefore, there is positive feedback activation between oxidative stress and inflammation in a vicious cycle in MS. A case–control study showed that biomarkers of oxidative stress were increased in patients with RRMS compared to healthy controls [75]. These findings proposed that oxidative stress can aggravate inflammatory reactions and contribute to more neuronal injury and progression of MS. Therefore, the use of antioxidants may hinder the development and progression of MS. Carlson and his colleagues proposed that antioxidants may play a beneficial role in human MS despite conflicting findings in animal MS model [76]. Evidence from preclinical and clinical trials showed that the use of antioxidant alpha lipoic acid reduces brain atrophy and improves the clinical course of MS [77]. It has been demonstrated that PPAR-α agonists have potent antioxidant effects and can ameliorate different neurodegenerative disorders, including AD [78]. PPAR-α agonist GW7647 inhibits lipid peroxidation, oxidative stress, and inflammation in mouse AD models [78]. An experimental study illustrated that fenofibrate improves antioxidant capacity and attenuates hyperglycemia-induced oxidative stress [79]. Oyagbemi et al. [79] revealed that PPAR-α agonist clofibrate attenuates oxidative stress in rats. Therefore, fenofibrate, through inhibition of oxidative stress and potentiating of endogenous antioxidant capacity, can mitigate MS pathogenesis.

Indeed, the propagation of neurodegeneration in MS and other neurodegenerative diseases is related to the reduction of neuroprotective brain-derived neurotrophic factor (BDNF) [61]. It has been illustrated that BDNF level is reduced in MS due to progressive neurodegeneration process [61]. A case control study on 22 MS patients compared to 19 healthy controls revealed that BDNF serum levels were reduced in MS patients compared to the controls [61]. However, a recent study observed that BDNF serum levels were not significantly reduced in MS patients compared to healthy controls [69]. A systemic review and meta-analysis involving 30 studies (689 MS patients and 583 healthy controls) revealed that BDNF serum level was reduced in MS patients compared to healthy controls [70].

Moreover, the fenofibrate neuroprotective effect can attenuate hippocampal insulin resistance and improve cognitive function in rats by increasing the expression of BDNF [48, 59, 127–130]. The neuroprotective effect of fenofibrate is mediated by increasing expression of BDNF in animal model studies [60]. In addition, fenofibrate upregulates the expression of hippocampal BDNF, which attenuates the neurodegeneration process in MS [60]. BDNF regulates microglia function toward trophic phenotype and prevents microglia-induced neurodegeneration [71, 131–134]. Notably, naturally derived phytoconstituents, including curcumin, cannabinoids, and genistein, reduce neurodegenerative diseases by increasing expression of BDNF [72]. These observations suggest that PPAR-α agonists such as fenofibrate can reduce MS neuropathology by improving the expression of BDNF.

Notoriously, autophagy plays a critical role in neurodegenerative diseases and could be beneficial in the early stage and detrimental in the late stage, as in AD and PD [90]. Autophagy is an essential intracellular degradative pathway to maintain normal cellular homeostasis by eliminating toxic proteins and injured organelles [115, 128, 135–138]. Defective autophagy in neurons induces the development of neurodegeneration [90]. Autophagy regulates adaptive and innate immunity, and autophagy abnormality triggers abnormal immune response [139–146]. Neuron function mainly depends on autophagy for its survival and homeostasis [147–149]. Defective autophagy contributes to the development and progression of MS. The autophagy process acts as a double-edged sword and could be protective or detrimental [150–155]. For example, increasing autophagy of T and B cells promotes the development of neuroinflammation, and inhibition of autophagy in this regard might be effective in treating MS. However, the induction of autophagy in neurons and glial cells improves the remyelination process [90, 91].

Interestingly, restoration of normal autophagy function in the early MS prevents the progression of disease severity [91, 156]. Autophagy-related genes are increased in T cells of MS brains [92]. These findings suggest a controversy regarding the role of autophagy in MS. Interestingly, restoration of normal autophagy function in early MS prevents the progression of disease severity [91]. Thus, targeting neuronal autophagy is important to enhance the remyelination process.

Different studies confirmed that fenofibrate improves the autophagy process [93, 158]. For example, fenofibrate attenuates cardiac injury in diabetic mice by increasing the expression of neuroprotective SIRT1 and autophagy function [93, 159–163]. In addition, fenofibrate attenuates acute kidney injury by regulating the autophagy process through the expression of adenosine monophosphate protein kinase (AMPK) [94, 164]. Both AMPK and SIRT1 activate the autophagy process [95, 165]. Therefore, fenofibrate through modulation of the autophagy process may attenuate the development and progression of MS.

Fenofibrate can attenuate MS neuropathology through modulation of different pathways, including oxidative stress, autophagy, mitochondrial dysfunction, inflammatory-signaling pathways, and neuroinflammation.

## Conclusion

MS is the most common inflammatory and demyelinating disease of the CNS. The fundamental pathophysiology of MS is the destruction of myelin sheath by immune cells or failure in the production of myelin. The characteristic feature of MS neuropathology is the formation of CNS plaques, which are multiple focal regions of demyelination distributed in the brain's white matter and spinal cords as well as in the deep grey matter and cerebral cortex. Notably, PPAR-α activator fenofibrate can attenuate the inflammatory reactions in MS by inhibiting the differentiation of Th17 and the expression of pro-inflammatory-signaling. Fenofibrate can reduce MS neuropathology by increasing the expression of BDNF. Fenofibrate, through inhibition of oxidative stress and potentiating of endogenous antioxidant capacity, can mitigate MS pathogenesis. Appropriate doses of fenofibrate could be effective against MS through modulation of mitochondrial dysfunction, autophagy process, inflammatory-signaling pathways, and neuroinflammation. Fenofibrate can reduce MS neuropathology through modulation of different pathways, including oxidative stress, autophagy, mitochondrial dysfunction, inflammatory-signaling pathways, and neuroinflammation. Therefore, preclinical and clinical studies are warranted to elucidate the precise role of fenofibrate in treating MS.

## Data Availability

All data are available in the manuscript.
